# Data Merging of AE Sensors with Different Frequency Resolution for the Detection and Identification of Damage in Oxide-Based Ceramic Matrix Composites

**DOI:** 10.3390/ma13204691

**Published:** 2020-10-21

**Authors:** Nicolas Guel, Zeina Hamam, Nathalie Godin, Pascal Reynaud, Olivier Caty, Florent Bouillon, Aude Paillassa

**Affiliations:** 1 IRT Saint-Exupéry, Esplanade des Arts et Métiers, F-33405 Talence CEDEX, France; aude.paillassa@irt-saintexupery.com; 2 INSA Lyon, MATEIS, University of Lyon, CNRS UMR-5510, 7 avenue Jean Capelle, F-69621 Villeurbanne CEDEX, France; zeina.hamam@insa-lyon.fr (Z.H.); pascal.reynaud@insa-lyon.fr (P.R.); 3 University of Bordeaux, LCTS, CNRS UMR-5801, 3 allée de la Boétie, F-33600 Pessac, France; caty@lcts.u-bordeaux.fr; 4 Safran Ceramics, Rue de Touban, F-33185 Le Haillan, France; florent.bouillon@safrangroup.com

**Keywords:** ceramic matrix composites, acoustic emission, sensor effect, damage mechanisms, feature-level fusion

## Abstract

In this paper, acoustic emission data fusion based on multiple measurements is presented for damage detection and identification in oxide-based ceramic matrix composites. Multi-AE (acoustic emission) sensor fusion is considered with the aim of a better identification of damage mechanisms. In this context, tensile tests were conducted on ceramic matrix composites, fabricated with 3M™ Nextel™ 610 fibers and aluminosilicate matrix, with two kinds of AE sensors. Redundant and complementary sensor data were merged to enhance AE system capability and reliability. Data fusion led to consistent signal clustering with an unsupervised procedure. A correlation between these clusters and the damage mechanisms was established thanks to in situ observations. The complementarity of the information from both sensors greatly improves the characterization of sources for their classification. Moreover, this complementarity allows features to be perceived more precisely than using only the information from one kind of sensor.

## 1. Introduction

Ceramic matrix composites (CMCs) are planned to be inserted into a new generation of civil aircraft engines. Their excellent thermomechanical properties and their low density are suitable for the requirements of hot components. In parallel with SiC-based CMCs, oxide-based CMCs have been developed to replace metallic rear parts of the engine. Indeed, non-oxide ceramic composites suffer from limits at elevated temperatures in terms of oxidation resistance. Oxide–oxide ceramic matrix composites [1,2,3,4,5,6] have been developed due to their promising high-temperature performance; they offer good stability against corrosive and oxidative atmospheres. Moreover, in comparison with SiC-based CMCs, oxide composites have been produced with simpler and cheaper manufacturing techniques. These CMCs contain oxide-based constituents in the fibers, such as alumina-based Nextel™ 610 and in matrices (e.g., alumina, YAG zirconia, mullite/SiOC). They are characterized by porous matrices without interphase between the fiber and matrix. The manufacturing process generates several heterogeneities at different scales in the material such as macroporosity, shrinkage cracks, intra-tow porosity and microporosity [7,8,9,10,11].

In this context, damage behavior of oxide-based CMCs is a key factor for the durability and reliability of components in service. Therefore, it is essential not only to characterize this damage behavior but also to identify the different damage mechanisms. To address this issue, acoustic emission (AE), which allows real-time damage monitoring, is a well-adapted technique for diagnosis and prognosis [12,13,14,15,16]. AE signals are generated from the release of energy originating from sources located within the material. For composite materials, several damage mechanisms act as AE sources such as matrix cracks, fiber breaks, fiber/matrix debonding and delamination. Identifying AE signatures of damage mechanisms is an established field [10,16]. The investigation of the collected data allows for identifying the different sources of damage and to determine the kinetics of the different degradation mechanisms during the lifetime.

Usually, piezoelectric sensors are applied directly on the samples’ surface to capture these elastic waves generated by damage mechanisms. Most of the time, acoustic emission is collected from resonant sensors due to their good sensibility. However, waveform characterization is strongly dependent on sensor features [17,18,19]. Therefore, the effects of sensors’ types are still a challenge. Undeniably, the measurement process provides observations that contain characteristics both from the damage mechanism and from the measurement process. The measurements recorded from sensors involve information incompleteness on the damage mechanism. It can only obtain partial information and therefore cannot get accurate information about the damage. Several studies have shown that using one type of sensor has many limitations in identifying acoustic signatures [17,18,19]. This emphasizes the interest of using multiple data sources and data fusion.

The aim of multi-sensor data fusion is to improve the quality and accuracy of collected information. The result is consequently enhanced in comparison with data collected by an individual sensor. Multi-sensor fusion describes the synergistic application of different homogeneous or inhomogeneous sensors to perform a given measuring task. Techniques to combine or fuse data are drawn from a diverse set of disciplines such as digital signal processing, statistics, artificial intelligence and pattern recognition. Sensor fusion or data fusion is defined as the data integration of multiple sensors to enhance the decision as well as to increase the reliability of the interpretation [20,21,22,23,24,25]. In this context, it is important to merge multiple sensors’ data without elimination of the relevant data. Therefore, multi-sensor data fusion can be defined as the process of combining data from several sensors so that the damage identification may benefit from all available sensor information. It is widely accepted that data can be combined at three levels: data, features and decisions [22]. Due to its simplicity, feature-level fusion is widely used. The use of features-based methods is recommended if the measurement data contain enough distinguishable and easily detectable features, which is the case for AE signals. Different feature vectors extracted from the same source always reflect the different characteristics of sources. A better description of damage mechanisms with feature enhancement can improve classification results.

To address the above limitations on the effects of AE sensors, this paper proposes a multi-AE sensor data fusion in order to monitor the damage kinetics and to identify damage mechanisms that occur in oxide-based CMCs. Information from two kinds of resonant sensors with complementary frequency range is considered in this paper. This configuration is complementary since sensors do not directly depend on each other. They can be combined in order to give a more complete image of the damage mechanism. Complementary sensors can help to resolve the problem of incompleteness, and they consist in the fusion of two images of the same damage mechanism taken from different viewpoints. Nevertheless, this configuration is competitive, as each sensor delivers an independent measurement of the same damage mechanism and the redundant information can be fused by evaluating the mean of each feature. In this context, all sensors contribute equally to the fusion result. The aim of the competitive fusion is to reduce the measurement uncertainty and to avoid measurement errors.

In this study, mechanical tests at room temperature were realized on oxide-based CMCs with a cross-weaved reinforcement. The investigated material was a weak-matrix oxide–oxide CMC fabricated with 3M™ Nextel™ 610 fibers and aluminosilicate matrix. Tensile tests were performed in the direction of fibers (0/90° tests). In situ mechanical tests were performed through SEM and synchrotron µ-tomography. Two kinds of AE sensors were used to obtain information in several frequency domains. All the waveforms were collected during the test for each sensor. The analysis was based on the descriptors calculated on the recorded waveforms during a specific post-processing phase. The rule-based method for the feature fusion is the linear weighted fusion. Data from the two sensors were fused in order to have global information from each source. Features from the two kinds of sensors are pooled into a single set. The outline of this paper is as follows: the experimental procedure and the acoustic emission analysis are first detailed in Section 2. The methodology used for data fusion on the feature level is also introduced in this section. Experimental results are discussed in Section 3. The paper ends with the conclusions and future work.

## 2. Experimental Procedure and Acoustic Emission Analysis

### 2.1. Description of the Material

**Figure 1:**
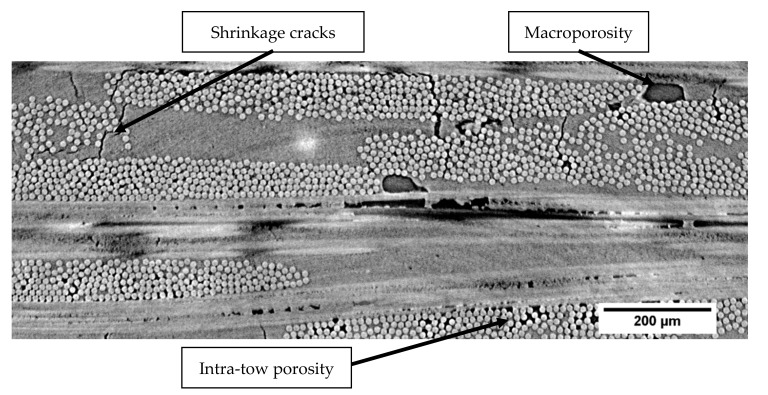
2D µ-tomographic reconstruction of oxide-based ceramic matrix composite (N610/Aluminosilicate) at the initial stage. Shrinkage cracks are mainly located in matrix area, and macroporosity is situated between plies.

The studied material was an oxide-based CMC with alumina fibers (Nextel™ 610 – 3M™) manufactured by Safran Ceramics group (Bordeaux, France). Fibers are aggregated in tows that are weaved in 8-harness satin (8HS) plies. The material is composed of 12 plies embedded in a microporous aluminosilicate matrix. The 12 plies of single weave fabrics contain fibers in 0°/90° orientations. The matrix is obtained from a partial sintering of ceramic powder. The matrix microporosity allows crack deviation on fiber-matrix interfaces in order to obtain a damage-tolerant material. Matrix infiltration through the fibrous structure induces porosities between (macroporosity) and inside the plies (intra-tow porosity). Due to the sintering process, shrinkage cracks were initially present in the material (Figure 1). Specimens have a fiber volume ratio between 45% and 50%.

### 2.2. Tensile Test and Acoustic Emission Recording Procedure

**Figure 2:**
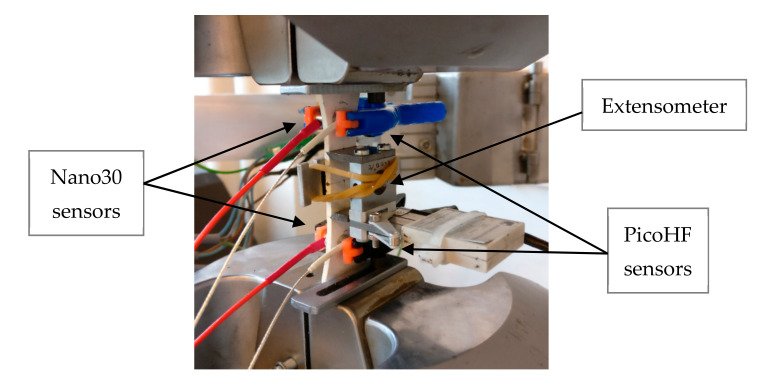
Multi-sensor acoustic emission (AE) monitoring during macroscopic tensile test.

Five tensile tests were done at room temperature. Displacement-controlled tests were done until specimen fracture. Strain was recorded with a 25 mm gauge length extensometer. Figure 2 shows the experimental set-up with the AE sensors. Mechanical tests were monitored with two kinds of resonant sensors operating at different frequency ranges (Nano30 and PicoHF sensors, MISTRAS Group, Paris, France). During the mechanical tests, the distance between the sensors was 50 mm. Sensor-material coupling was achieved with vacuum grease. Pre-amplification of 40 dB and band-pass filtering of 20–1200 kHz was performed with preamplifiers. The acquisition threshold was set to 40 dB. Preamplified signals were recorded with Mistras PCI2 data acquisition system (MISTRAS Group, Paris, France). The waveforms were recorded, and the hits were extracted during a post-processing step. Wave propagation speed was previously determined with Hsu Nielsen sources (pencil lead breaks) and set to 6800 m·s^−1^ in the fibers’ axis for the undamaged state.

**Figure 3:**
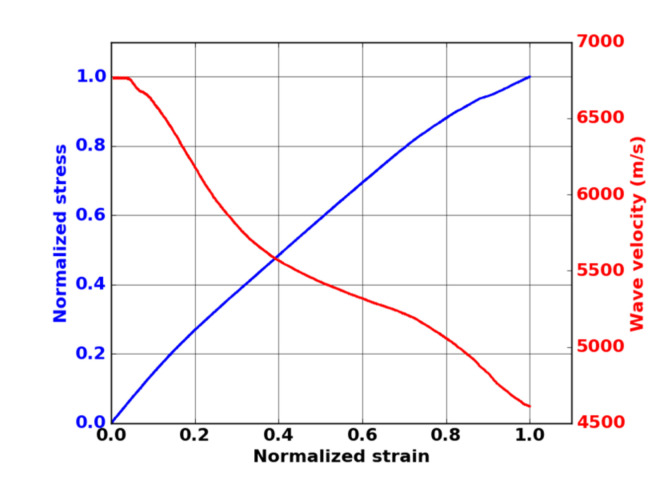
Evolution of the wave velocity as a function of strain during tensile test in fiber orientation on oxide–oxide composite.

For the location of AE signals along the gauge length, it is important to take into account the evolution of wave velocity during the mechanical test due to damage. The velocity of an extensional wave in a thin plate is proportional to the square root of the elastic modulus E of the material. As proposed by Morsher [26], the initial modulus during unloading $E\left(\varepsilon \right)$ was measured during a monotonic tensile test with successive unloading/reloading sequences. Hence, hysteresis loops were obtained at different strains, and the velocity $Ce\left(\varepsilon \right)$ was then determined by using Equation (1):

(1) $$\frac{Ce\left(\varepsilon \right)}{Ce_{0}}=\sqrt{\frac{E\left(\varepsilon \right)}{E_{0}}}$$

where $Ce_{0}$ and $E_{0}$ represent, respectively, the velocity and the elastic modulus in the undamaged state, and $Ce\left(\varepsilon \right)$ and $E\left(\varepsilon \right)\;$ are, respectively, the velocity and the elastic modulus under a strain ε. Figure 3 shows the evolution of the wave velocity versus strain. Just before the final rupture, the estimated velocity on the composite was equal to 4600 m·s^−1^ (Figure 3). Thus, the decrease in wave velocity is not negligible. To perform reliable assessments of the sources’ positions, this variation as a function of strain was taken into account.

### 2.3. Characterization of the Sensor with an Acousto-Ultrasonic Card

**Figure 4:**
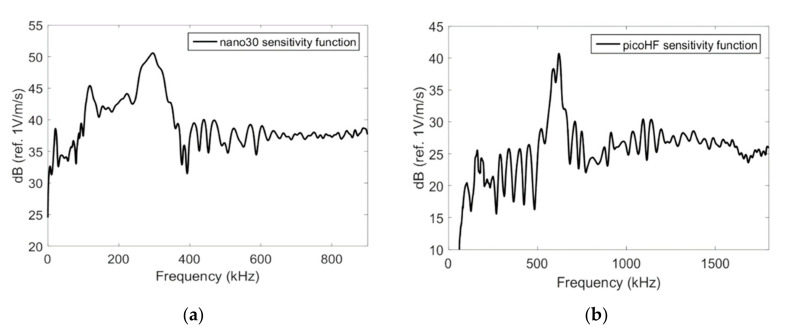
Response in reception on steel block for the Nano30 (**a**) and PicoHF (**b**) sensors obtained with the reciprocity method.

Figure 4 shows the Rayleigh wave reception sensitivity of Nano30 and PicoHF sensors obtained with the reciprocity method [19,27,28,29]. The determination of the sensitivity requires three transducers alternatively working as transmitters and receivers. The sensitivity in the reception was measured on a steel block for Rayleigh waves. Nano30 sensors have higher sensitivity and lower frequency range. These two sensors showed a good sensitivity for the frequencies from 200 to 400 kHz for Nano30 sensor and 500 to 1850 kHz for PicoHF sensor.

**Figure 5:**
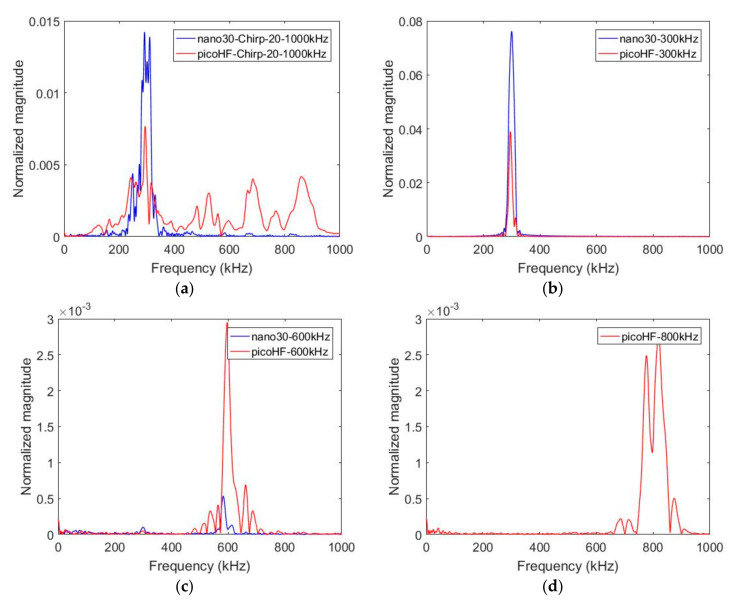
Fast Fourier Transform (FFT) of the recorded signals with Nano30 sensor and PicoHF sensor for emitted signals at different frequencies (**a**) chirp, (**b**) 300 kHz, (**c**) 600 kHz, (**d**) 800 kHz generated by an acousto-ultrasonic card. The input signal is generated with a specific frequency, amplitude of 5 volts and a rise time of 20 µs. (Propagation distance of 20 mm, epoxy material propagation, actuator Micro80 sensor). (**a**) Chirp, (**b**) 300 kHz, (**c**) 600 kHz and (**d**) 800 kHz.

In order to characterize the response of each sensor for the same source, tests with an acousto-ultrasonic card were also done on polymer material. The specimen was a 2 mm-thick plate of epoxy with a surface of 140 × 140 mm^2^. This technique involves exciting a transducer, a Micro80 sensor (MISTRAS Group, Paris, France) in this study. An ARB card (ultrasonic generator ARB 1410-150, MISTRAS Group, Paris, France) delivered the excitation signal. Burst-type signals were was (amplitude 5 V, frequency range from 20 to 1000 kHz, rise time 20 µs). The generated excitation is achieved with different frequency contents between 100 and 1000 kHz and with a chirp in the same frequency range. The signal was transferred as a stress wave into the plate by the Micro80 sensor. The transducer used as transmitter was located in the middle of the sample. Acoustic waveforms with specific frequencies were generated. The two receivers (Nano30 and PicoHF) were situated on a straight line at the same distance from the transmitter (20 mm). The acquisition parameters were those set for AE monitoring. Figure 5 shows the results obtained with a chirp and with specific frequency content for the excitation signal (300 kHz, 600 kHz and 800 kHz). Although the sensitivity function of the sensor in emission (Micro80) plays an important role in the emission, these experiments allow comparing reception responses of the sensors for the same source. Moreover, the propagation medium alters the source signal. These aspects were not taken into account in this study. These measurements are mainly used to determine the effect of the sensor for the same source and the same propagation medium.

With the chirp, PicoHF sensors exhibited complex frequency spectra from 100 to 900 kHz, whereas Nano30 has a narrowband response with no significant response to frequencies above 400 kHz (Figure 5a). This point was confirmed with the mono-frequency excitation. With Nano30 sensors, it is not possible to properly detect frequency content above 400–500 kHz (Figure 5c). If the source mechanisms contain frequencies above 400–500 kHz, only PicoHF sensors can detect these mechanisms correctly. For an excitation signal of 800 kHz, there was no response for the Nano30 sensor (Figure 5d). On the other hand, the Nano30 sensor had the best reproduction of content below 400 kHz (Figure 5b). These results confirm the good complementarity of the two types of sensors.

### 2.4. Pre-Processing of the Waveforms and Features Extraction

**Figure 6:**
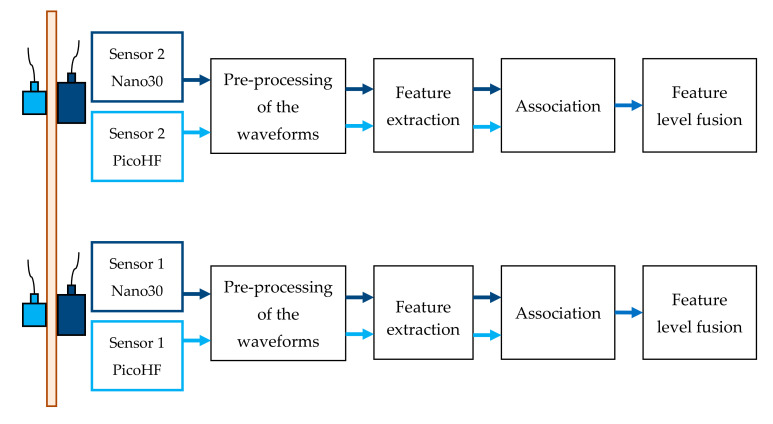
Processing flowchart for features-based signal fusion.

The whole procedure is summarized in Figure 6. This figure is a flowchart of the framework to study damage and identify AE signature for oxide-based CMCs. The waveform pre-processing step was accomplished after AE signal collection. The aim of this step is to remove useless information and improve the waveform aspect in order to characterize them. The process results from previous works [30]. Waveform preprocessing was performed on waveforms directly acquired from sensors. Consecutively to pre-trigger deletion, tail cutting was achieved from an energy criterion to remove the end of the signal. Discretized energy of each waveform was determined from temporal sliding window equal to 10 μs. For each point in the waveform, the cumulative energy computed from the beginning was also calculated. If the energy in the sliding window following a point in the signal was lower than 0.1% of the cumulative energy from the beginning of the signal, the starting point of the window was considered at the end of the signal. Shape-preserving interpolation resampling and wavelet denoising were successively performed on waveforms. The denoising was carried out following a wavelet decomposition of the waveform at level 3 from a Symmlet 8 mother wavelet.

**Figure 7:**
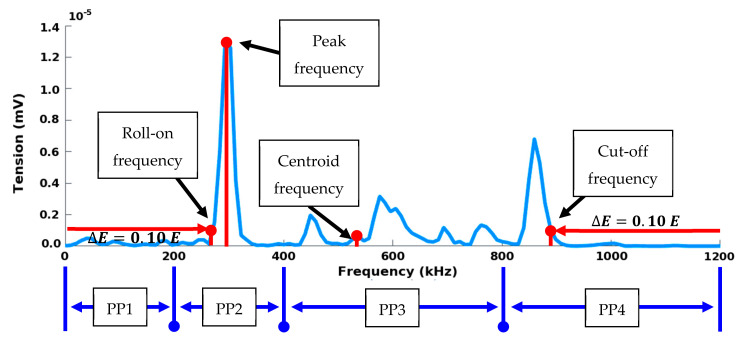
Descriptors calculated in the frequency domain on a digitized AE signal.

Thus, each waveform was turned into a compact representation through a set of 25 features. Descriptors (Table 1) were calculated from pre-processed waveforms in temporal domain and frequency domain (Figure 7) using Fast Fourier Transform (FFT). Temporal features can be extracted from the temporal evolution of the signal such as amplitude, rise time, duration, energy, etc. Besides conventional descriptors, calculated descriptors were implemented from former AE analysis on composite materials and corrosion detection [30]. The opening frequency f_op_ or the roll-on frequency is such that the interval ]0; f_op_] contains ∆E= 10% of the energy E of the complete spectrum. Thus, it gives information on the presence of components at low frequencies. The cut-off frequency f_cut_ (or roll-off frequency) is such that the interval ]0; f_cut_] contains E−∆E= 90% of the energy of the complete spectrum.

The partial powers, denoted $\;PP_{ikHz}$, have been defined according to the sensitivity function of each sensor (Figure 3). For both sensors, the frequency bands of each $PP_{ikHz}$ are as follows: 0–200, 200–400, 400–800 and 800–1200 kHz. Furthermore, the spectral characteristics of the waveforms were analyzed in time/frequency domain with the Smoothed Pseudo Wigner-Ville Distribution (SPWVD).

In order to compare the sensibility of the sensors during the test, the following ratio denoted R was calculated for each source detected by the two types of sensors (Equation (2)).

(2) R=PP2nano30−200−400 kHz∗Enano30Enano30¯PP3picoHF−400−800 kHz+PP4picoHF−800−1200 kHz∗EpicoHFEpicoHF¯

where $PP_{i\;sensor-kHz}\;$ is the partial power in the main frequency range of each sensor, $E_{sensor}$ is the recorded energy of the signal and $\bar{E_{sensor}}$ is the average signal energy recorded during the test for each sensor. This ratio allows the estimation of the frequency balance of each signal:

- if R is higher than 1, the content of the signal is mainly low-frequency, i.e., detected by Nano30 sensors,
- if R is lower than 1, the content of the signal is mainly high-frequency, i.e., detected by PicoHF sensors.

### 2.5. Damage Indicator

**Figure 8:**
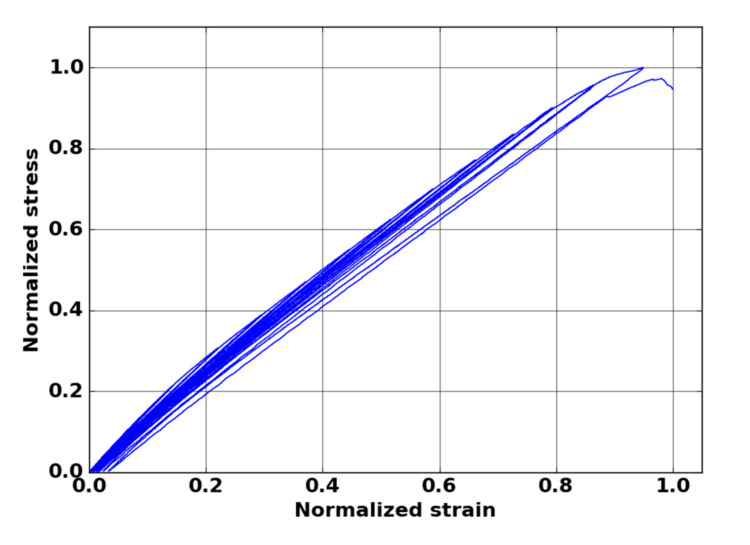
Stress versus strain normalized curve obtained from cycled tensile test in fiber orientation of oxide–oxide composite.

Two damage indicators were defined: on one hand, a mechanical indicator based on the evolution of the secant elastic modulus and, on the other hand, an indicator linked with the severity of AE activity. The secant elastic modulus was calculated from the strain–stress curve. For this kind of material, we achieved two mechanical tests with unloading–reloading cycles (Figure 8). The mechanical behavior showed very low residual strain, even close to the failure. In this case, the secant elastic modulus was close to the theoretical modulus $E_{the}\;$ obtained by neglecting the residual strain. Thus, the modulus $E_{the}\;$ can be calculated directly from monotonic tensile tests by the slope between the points of the mechanical curve and the origin.

The second damage indicator is based on the determination and the investigation of the acoustic energy released. In this study, the severity (Equation (3)) introduced by Fowler [31] was used in order to estimate the material degradation.

(3) $$\;S_{e}=\;\frac{1}{J}.\overset{J}{\underset{i}{\sum }}\;E_{i}$$

where $S_{e}$ is the energy-based acoustic severity, J is the number of most energetic signals retained for the calculation (set to 10) and E_i_ is the energy of the ith most energetic signal.

### 2.6. Signal Alignment and Feature Level Fusion

It is necessary to determine which signals represent observations of the same AE source. Data association is used to associate measured data from multi-sensors and create pairs by comparison of the acquisition time. Sources recorded with these two kinds of sensors (located at the same position along the gauge length) are associated using acquisition time. Signals are considered associated if they are detected in a temporal window lower than 5 µs. This interval is lower than the time of wave propagation between the sensors (50 mm). Associated signals represent 80% of the signals recorded with PicoHF sensors. Feature extraction is processed separately for each waveform. The first step consists of obtaining the most informative data from the two kinds of sensors. Then the features are all merged together. In this study, the multi-AE sensor data were commensurate, i.e., the sensors were measuring the same physical phenomena. Therefore, the raw data could be directly combined. In this work, the feature-level fusion was chosen. To enhance the contrast, adding and multiplication can be used [32]. Features fusion rule is based on the average value. D’Costa and Sayered [33] showed that when measurements are correlated and independent, data averaging yields the optimal solution.

Due to complementary information collected from the two kinds of sensors, calculated descriptors from both sensors were merged using mean value (Equation (4)). All sensors contributed equally to the fusion results. Energetic descriptors were obtained from root mean square (Equation (5)).

(4) $$d_{merged}^{i}=(d_{Nano30}^{i}+d_{PicoHF}^{i})/2$$

(5) $$d_{merged}^{i}=\;\sqrt{\left(d_{Nano30}^{i}{}^{2}+d_{PicoHF}^{i}{}^{2}\right)/2}$$

where:

- $d_{\mathrm{merged}}^{i}$: descriptor i calculated from the two kinds of sensors,
- $d_{\mathrm{Nano}30}^{i}$: descriptor i calculated from the waveform recorded with Nano30 sensor,
- $d_{\mathrm{PicoHF}}^{i}$: descriptor i calculated from the waveform recorded with PicoHF sensor.

### 2.7. Data Clustering: Unsupervised Approach

Data clustering was carried out from calculated signal characteristics [34,35]. Uncorrelated descriptors were selected with dendrogram classifier. By choosing a cutting height on the dendrogram equal to 0.3, seven groups of descriptors were created. The choice of relevant descriptors in the different groups is still an open question. Indeed, the choice of descriptors has an impact on the segmentation of the data. Several sets of descriptors were tested. The set of relevant descriptors was determined by testing different combinations and observation of the quality of the classification thanks to Davies and Bouldin [35] and silhouette [36] criteria. PCA analysis was applied to the selected descriptors to reduce the data dimension and to create new uncorrelated features. K-means clustering optimized by genetic algorithm operates several data partitions from 2 to 5. The algorithm stops when the change of the average Davies-Bouldin index value of the population is less than 10^−7^ over 50 generations. The genetic strategy is characterized by a high stability and a high performance [37]. The clustering was run 10 times for each set of descriptors and the more reproducible result was saved. Fundamental issues to be addressed in building a data fusion include which accuracy the data fusion process can realistically be achieved. All the signals recorded by both types of sensors or with the combined data were taken into account for the classifications. After the clustering, data with negative individual silhouettes were not taken into account for the analysis.

## 3. Results

### 3.1. Global Mechanical Behavior and Ability to Detect Early Damage

**Figure 9:**
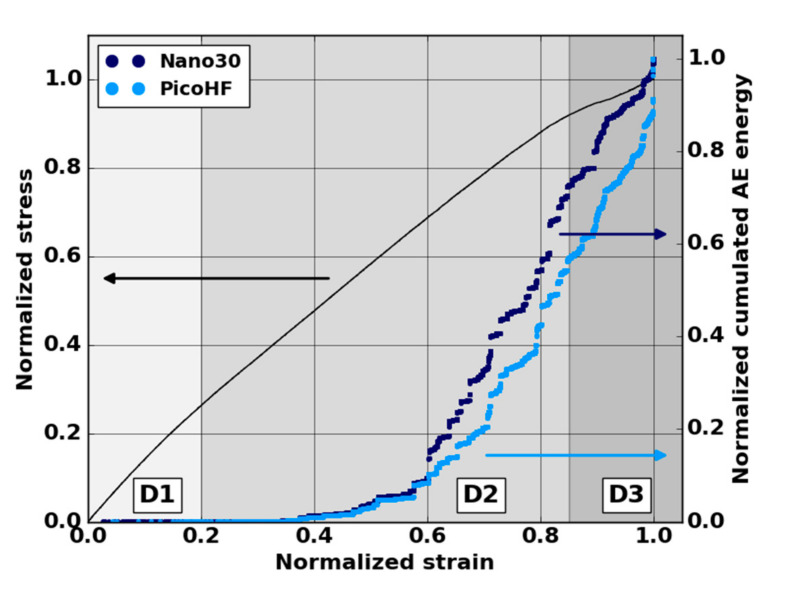
Stress-strain curve and global AE activity in terms of normalized cumulated AE energy during a macroscopic tensile test in the fiber orientation obtained from Nano30 and PicoHF sensors.

Following AE analysis is based on five macroscopic tensile tests in the fiber orientation to guarantee results repeatability. Mechanical behavior exhibited three domains (Figure 9): a first linear part, which ended at ε = 0.05 in normalized strain; a second linear part, which appeared up to ε = 0.7–0.85; and the last domain led to the macroscopic failure of the sample. The studied material exhibits more pronounced damaged behavior close to ultimate stress (ε > 0.8–0.9). Global AE analysis also revealed a significant increase in the acoustic energy for a normalized strain ε = 0.3–0.4 for both sensors (Figure 9).

**Figure 10:**
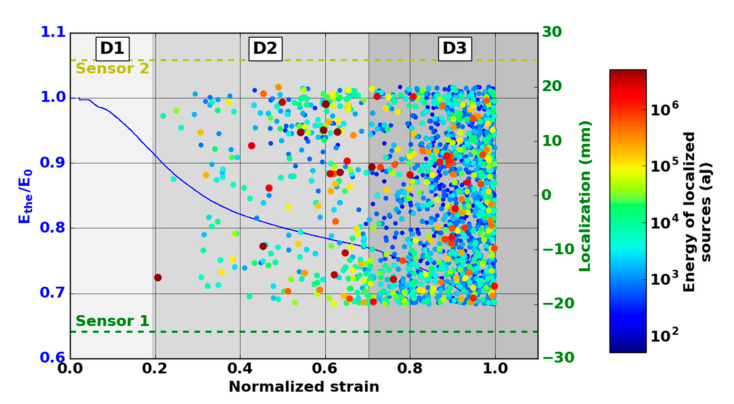
Evolution of the normalized modulus and the location of Nano30 AE signals versus strain during the macroscopic tensile test in the fiber orientation. (The size and the color of the dot are linked with the energy.)

The evolution of the modulus $E_{the}$ allows the identification of three phases in the damage evolution (Figure 10). The first domain, denoted D1, shows a reduction of the mechanical properties. The domain D2 starts with a slowdown in the decrease of the modulus. The third domain begins with the final significant decrease of the modulus. The transition between domains D1 and D2 was observed for a modulus $E_{the}\;$ between 90% and 95% of the Young modulus $\;E_{0}$. This transition was around ε = 0.2. The transition D2-D3 was obtained for a modulus $E_{the}$ between 74% and 76% of the Young modulus $\;E_{0}$. This transition was obtained for a normalized deformation near ε = 0.8.

A larger number of sources are detected with Nano30 sensors during mechanical tests. Nano30 sensors detected between 20% and 60% more signals than PicoHF sensors. It should be noted that more than 80% of the sources detected by PicoHF sensors were also detected by Nano30 sensors. The sources detected only by the Nano30 sensor were mostly low-amplitude sources. The detection of the first sources of AE was therefore later for PicoHF sensors. Because of the high attenuation of the material, which was certainly due to high porosity, only 15%–20% of the AE data were localized (Figure 10). Localized sources appeared in domain D2 and increased in domain D3. However, the most energetic sources were mainly recorded in the D2 domain. The locations evenly distributed over the entire useful area indicate diffuse damage. For both types of sensors, no sources were located during domain D1.

**Figure 11:**
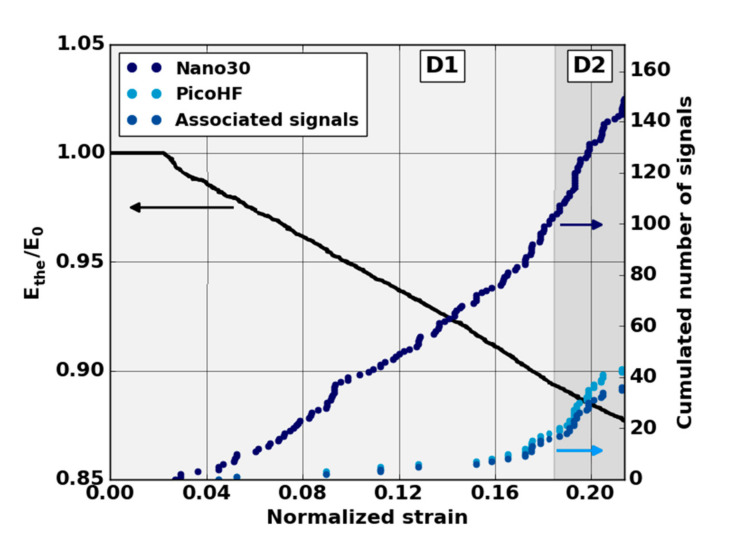
Evolution of the normalized modulus and cumulated number of AE signals recorded from each and both sensors versus strain during the domain D1 of macroscopic tensile test in the fiber orientation.

The absence of localized sources during the stage D1 (domain with a reduction of mechanical properties) led us to analyze all the recorded signals. The evolution of the cumulative number of recorded signals highlights the appearance of the first sources from the D1 domain mainly for the Nano30 sensors (Figure 11). It should be noted that the appearance of the first signals is correlated with the lowering of the theoretical modulus during the D1 domain. The first signals appeared around ε = 0.04 in normalized deformation for all tests. The mechanisms responsible for this degradation were detected by AE but were not sufficiently energetic to be localized. With regard to detecting the early stage of damage, the comparison of the two types of sensors shows that the Nano30 sensors are more sensitive than the PicoHF sensors. Nano30 sensors are more suitable to detect early damage.

### 3.2. Damage Evolution

**Figure 12:**
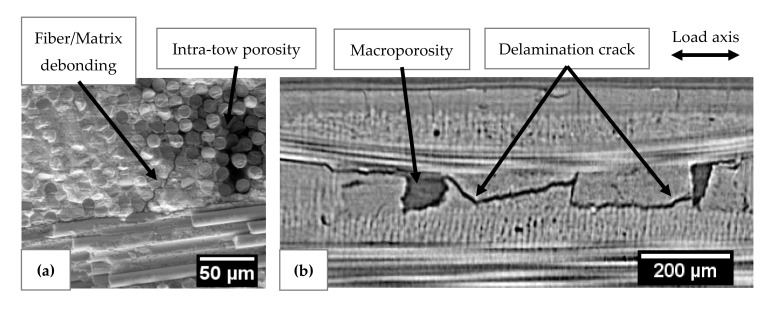
(**a**) In-situ SEM observation revealing intra-tow fiber/matrix debonding ($\sigma_{\mathrm{normalized}}$ = 0.2); (**b**) In-situ 2D µ-tomographic reconstruction showing crack delamination ($\sigma_{\mathrm{normalized}}$ = 0.7).

**Figure 13:**
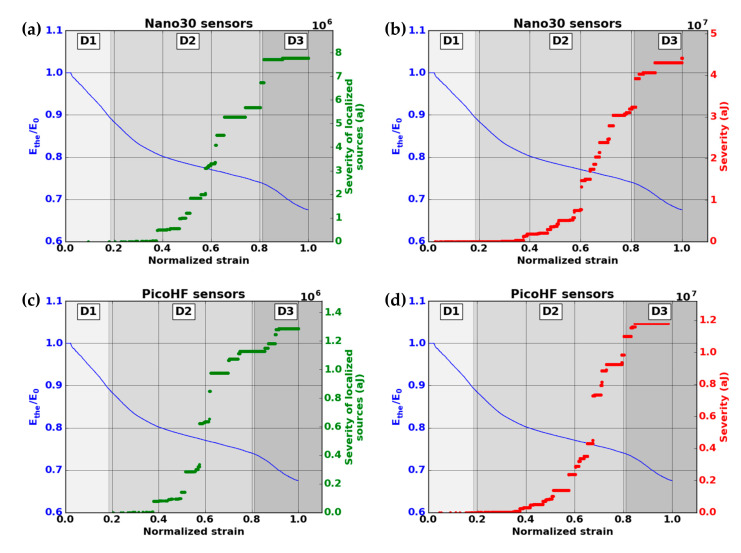
Evolution of the normalized elastic modulus and the severity versus strain during macroscopic tensile test in fiber orientation for (**a**) AE-localized signals by the Nano30 sensors, (**b**) for all the recorded signals by the Nano30 sensors, (**c**) AE-localized signals (PicoHF sensors) and (**d**) all the recorded signals (PicoHF sensors).

The microscopic observations reveal several damage mechanisms such as matrix cracking, fiber/matrix debonding and delamination crack (Figure 12). The severity of acoustic activity was evaluated to study the kinetics of the occurrence of critical damaged sources. The evolution of severity was characterized by jumps showing the appearance of highly energetic sources regardless of the type of sensor (Figure 13). To monitor the growth of damage, both types of sensors are relevant.

The severity study shows that a significant part of acoustic energy is contained in a limited number of signals. Since the severity was calculated with the 10 most energetic signals (J = 10), these 10 signals cover between 25% and 50% of the cumulative acoustic energy during a test. This proportion is higher than 75% if only localized sources are considered.

The damage initiating the decrease of mechanical properties in domain D1 was generated from low energy sources. This resulted in very low severity. The damage observed by microscopy reveals that smaller-scale damage is widely present in these materials such as matrix cracks and intra-tows debonding. This observation points to multi-cracking of the microporous matrix. The bridges between the partially sintered grains of the matrix are in the order of a hundred nanometers, so the release of energy from their cracking is assumed to be low. The decrease during D1 is due to damage perpendicular to the loading axis such as matrix cracks and intra-yarn debonding.

The very high-energy signals were mainly recorded during the second damage domain D2. In comparison with the evolution of the modulus $\;E_{the}$, this result may appear contradictory because the most critical damage occurred in stage D2 with the least significant reduction in mechanical properties. In regard to the fracture surfaces, it is assumed that these signals were associated with delamination phenomena. Since these large-scale damages occurred in planes parallel to the loading axis, they did not directly influence the mechanical properties determined using the stress–strain curves. Moreover, AE monitoring of X-ray Synchrotron tomography [38] confirms the link between the energetic signals with large inter-plies debonding that led to delamination of the material. Domain D2 is characteristic of a load transfer through the warp yarns, which caused a slowdown in the loss of mechanical properties. This stress recovery results in shear forces in the material leading to damage parallel to the loading axis such as inter-yarns debonding and then delamination.

A slight increase in severity reflecting the absence of more energetic sources was observed in domain D3. This domain was characterized by a drop in mechanical properties due to the breakage of the fibers contained in the warp yarns. These ruptures led to the macroscopic ruin of the material. Fiber breaks are associated with the D3 domain. Delamination, observed in D2, releases higher acoustic energy than fiber breaks. To monitor damage evolution during D2 and D3, the two kinds of sensors are suitable.

### 3.3. Waveforms Characteristics and the Combined Behavior of the Two Kinds of Sensors

**Figure 14:**
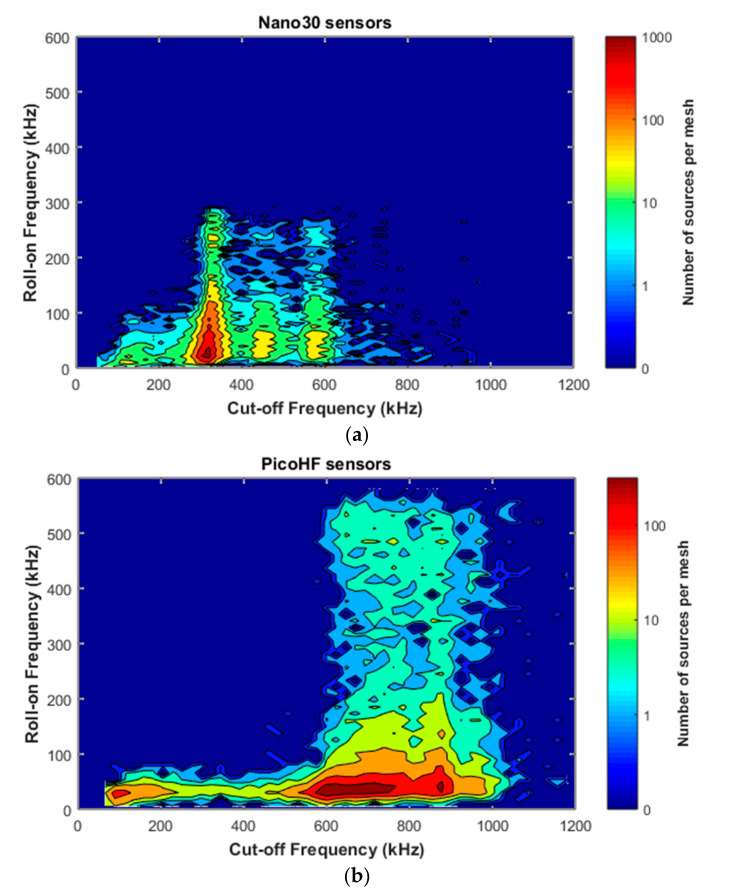
Representation of acoustic emission signals in the plane opening frequency and cut-off frequency; (**a**) Nano30 sensors, (**b**) PicoHF sensors—signals recorded during macroscopic tensile test in fiber orientation.

**Figure 15:**
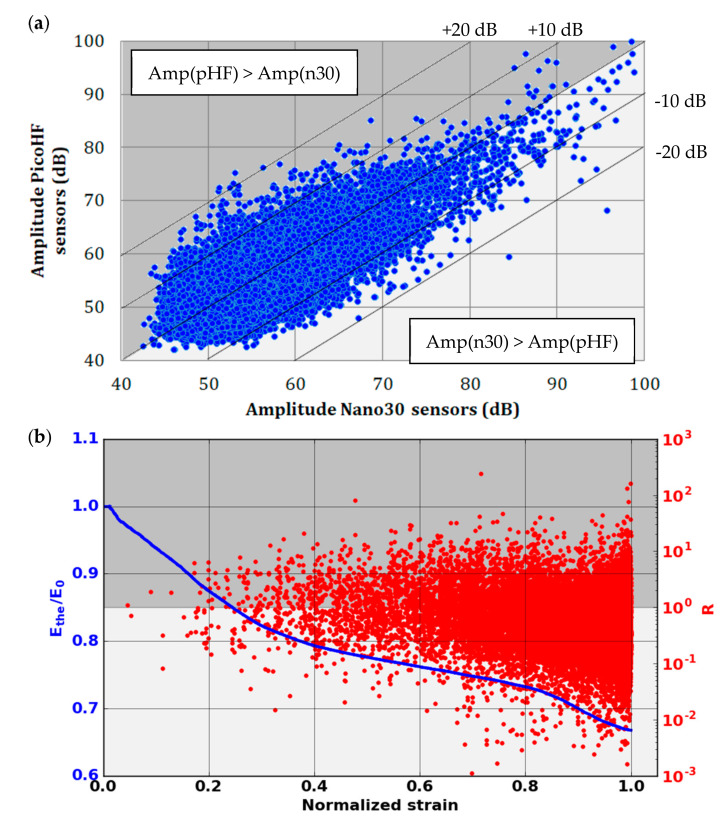
(**a**) Amplitude of the same signals recorded by Nano30 and PicoHF sensors, before the combination, in the plane Amp(Nano30) versus Amp(PicoHF) and (**b**) evolution of the coefficient R versus strain during the macroscopic tensile test in fiber orientation.

Due to the difference in sensitivity in the frequency domain for Nano30 and PicoHF sensors, signals recorded by PicoHF sensors revealed higher frequency content than Nano30 signals. Figure 14 illustrates the associated signals distribution in opening frequency versus cut-off frequency. The frequency centroid was higher for the signals recorded by PicoHF sensors. These results suggest that some of the signals generated during the tensile tests had high-frequency content that was not correctly detected by the Nano30 sensors. Figure 15a shows the amplitudes of the associated signal. Despite a better sensibility of Nano30 sensors, a consequent number of signals exhibited higher amplitude with PicoHF. This observation seems to be correlated with the main frequency of these signals (Figure 15b). The evolution of the R coefficient reveals a high-frequency enrichment of data recorded by PicoHF sensors (i.e., signals with very low R ratios) just prior to the final rupture. This result highlights that PicoHF sensors were more suitable towards the end of the test to characterize the sources.

### 3.4. Unsupervised Clustering and Labelling of the Clusters

**Figure 16:**
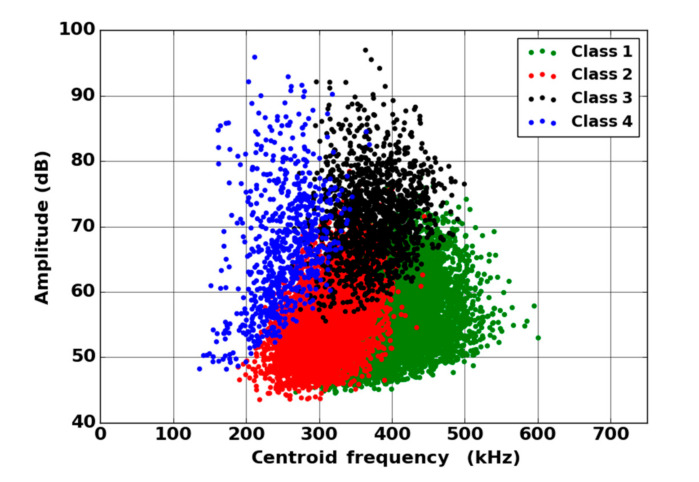
Result of the segmentation, in the plane Amplitude/Centroid Frequency, for the fused AE data recorded during a tensile test on oxide/oxide composite.

**Figure 17:**
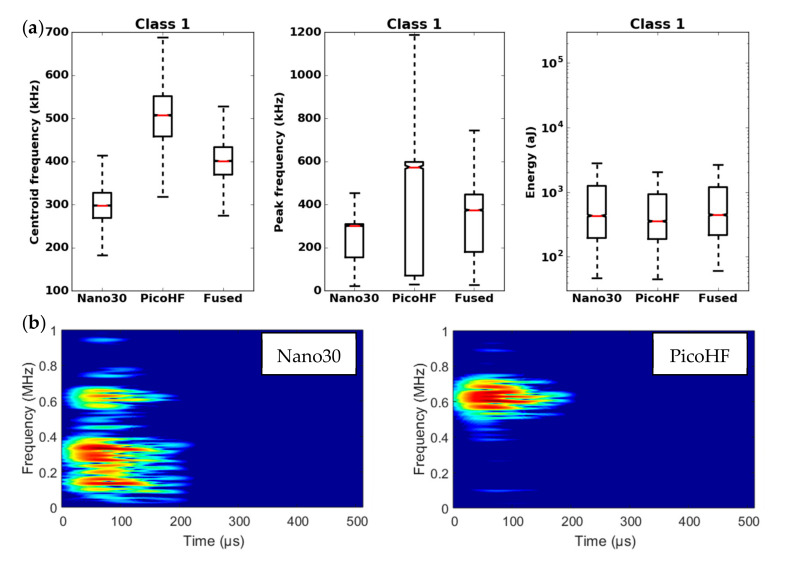
Results of the classification with an unsupervised classification based on fused features. (**a**) Characteristics of class 1 (frequency centroid, peak frequency and energy) viewed by the Nano30 sensor, PicoHF or with merged descriptors. (**b**) Representation in time/frequency domain with the Smoothed Pseudo Wigner-Ville Distribution (SPWVD) of the same AE signal recorded by Nano30 sensor and PicoHF sensor for class 1.

**Figure 18:**
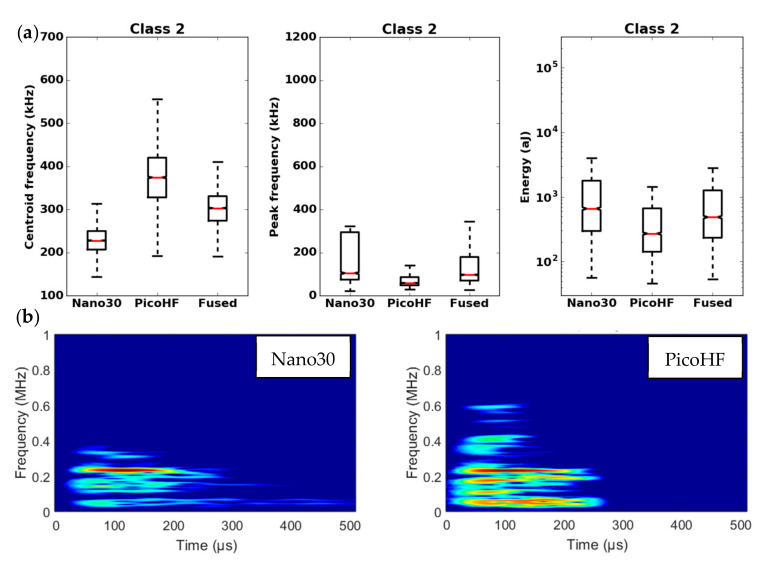
Results of the classification with an unsupervised classification based on fused features. (**a**) Characteristics of class 2 (frequency centroid, peak frequency and energy) viewed by the Nano30 sensor, PicoHF or with merged descriptors. (**b**) Representation in time/frequency domain with the SPWVD of the same AE signal recorded by Nano30 sensor and PicoHF sensor for class 2.

**Figure 19:**
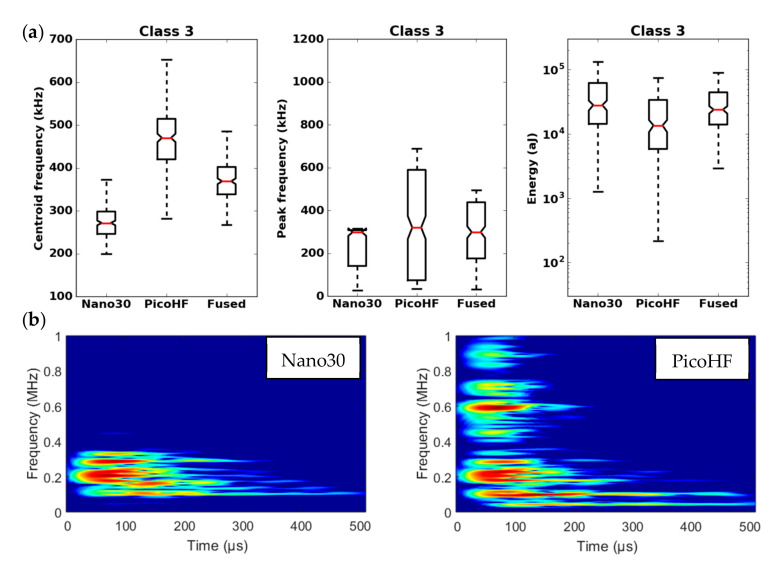
Results of the classification with an unsupervised classification based on fused features. (**a**) Characteristics of class 3 (frequency centroid, peak frequency and energy) viewed by the Nano30 sensor, PicoHF or with merged descriptors. (**b**) Representation in time/frequency domain with the SPWVD of the same AE signal recorded by Nano30 sensor and PicoHF sensor for class 3.

**Figure 20:**
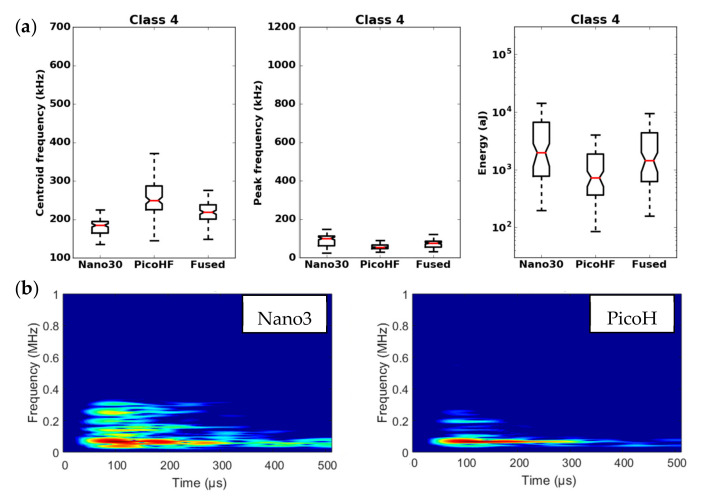
Results of the classification with an unsupervised classification based on fused features. (**a**) Characteristics of class 4 (frequency centroid, peak frequency and energy) viewed by the Nano30 sensor, PicoHF or with merged descriptors. (**b**) Representation in time/frequency domain with the SPWVD of the same AE signal recorded by Nano30 sensor and PicoHF sensor for class 4.

By means of the dendrogram, for each group of correlated descriptors, the descriptors with the greatest relevance to the analysis were kept. Seven uncorrelated fused descriptors were retained for each unsupervised classification: four in the time domain (duration, energy, rise time and rise angle); two in the frequency domain (centroid frequency and spectral spread); one combined descriptor (amplitude–centroid frequency ratio). A Principal Component Analysis (PCA) was performed with the descriptors mentioned above in order to obtain more accurate partitions. The first classification of AE signals is based on features extracted from each sensor without fusion. Whatever the type of sensors, classification of signals coming from tensile tests gives two classes, with one minor class that contains the major part of the acoustic energy. In this case, without descriptors fusion, for the two kinds of sensors, the segmentation consisted of a cluster that contained few signals (less than 5%) and another cluster composed of the remaining signals. However, these signals were very energetic and contained more than 75% of the acoustic energy. Moreover, the remaining 95% of the signals were in the additional class and therefore were associated with several damage mechanisms. This classification does not make it possible to identify the different mechanisms of the observed damage. The same procedure with the fused features led to an optimal solution with four classes. With the fused features, due to its repeatability and good signal assignment in each class based on the Silhouette values, the analysis of AE data was based on the four-class partition (Figure 16). The input data for classification appears to be more robust. Fusion increases the breadth of the waveform description. The specific characteristics of the classes were confirmed by the time-frequency distributions obtained by the two types of sensors (Figure 17, Figure 18, Figure 19 and Figure 20). These figures display boxplots of three descriptors (Centroid frequency, Peak frequency and Energy) calculated on the same signals recorded with the Nano30 and PicoHF sensors and classified with the fused descriptors. Boxplots indicate the median value by the mean of the red horizontal line, the lower quartile Q1, the upper quartile Q3, the maximum and the minimum values. A notch is added to show the 95% confidence interval for the median value. These figures also show the time/frequency representation for the same signal recorded by the two kinds of sensors. For the representation, signals chosen have descriptors representative of their class close to the median value.

**Figure 21:**
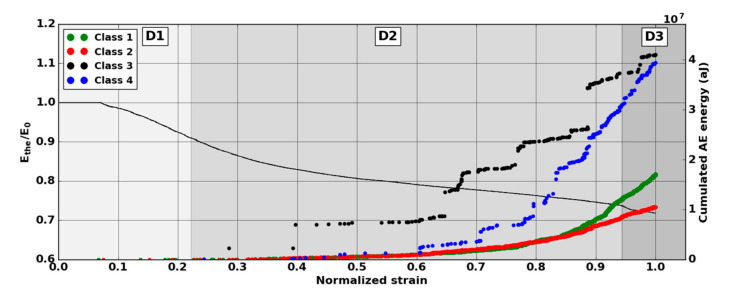
Kinetics of the several classes versus normalized strain during macroscopic tensile test in the fiber orientation. Results of the classification with an unsupervised classification based on fused features.

It should be noted that the frequency content of classes 1 and 3 signals exceeded 500 kHz, which was mainly detected by the PicoHF sensor. The signals associated with classes 2 and 4 had frequency contents mainly below 400 kHz. Frequency content below 200 kHz above 200 µs was also observed for classes 3 and 4 (Figure 19b and Figure 20b). The signals characteristics of each class as well as the evolution of these classes (Figure 21) during the test make it possible to propose a label, in accordance with the various microstructural observations: traction with observation using a microscope, in situ traction under MEB and under synchrotron.

Classes 3 and 4 contained the most energetic signals that contribute to the increase in acoustic severity. The location of these sources is correlated with the failure zones. During tensile tests in the fiber axis, these classes appeared in the D2 domain, with in particular a strong localization of these sources in this domain. Classes 3 and 4 were therefore associated with delamination phenomena, which were the most important damages observed during in situ monitoring. However, there were differences between classes 3 and 4. Class 3 was characterized by higher frequency content than class 4. For class 3, the PicoHF reveals a rich frequent content up to 1 MHz (Figure 19b). The PicoHF sensor is more suitable for the characterization of these signals and, the addition of this sensor allows identifying this class. The class 4 signals were characterized by low-frequency content, mainly lower than 200 kHz, regardless of the sensor type, around 100 kHz. These signals indeed had low frequencies, and certainly none of the used sensors are really suitable to characterize these signals. Class 3 appeared before class 4. The beginning of domain D2 was characterized by inter-yarns debonding. Class 3 was associated with the occurrence of delamination of slightly damaged plies and class 4 with the propagation of delamination.

Classes 1 and 2, which contained low energy signals, were observed simultaneously at the beginning of the test. Class 1 exhibited very high-frequency content (Figure 17 and Figure 18). Through the PicoHF sensors, the detected frequencies were around 600 kHz to 1 MHz. For the Nano30 sensors, the main frequency was around 300 kHz. Nevertheless, consequent frequency content was recorded around 600 kHz. This frequency content, outside of the Nano30 typical frequency response, shows that high-frequency content detected by these sensors is certainly more relevant than the main frequency content (around 300 kHz). Having multiple sensors sensitive to different frequencies enhances the identification of damage mechanisms. The second class was characterized by a main frequency content centered on 250 kHz with both sensors. In this case, the Nano30 sensors seem more suitable to characterize these signals. The decrease of mechanical properties was generated by intra-weft yarn debonding as well as matrix cracking and opening of shrinkage cracks. The apparent decrease in mechanical properties in D1 range is correlated with lower acoustic activity. Class 1 was composed of low-energy sources and with the highest-frequency content. This class appeared first. Sources in this class were associated with matrix cracking. The fine grain size of the matrix induced small cracks. Class 2 sources were also low in energy but with a lower frequency content. This class appeared quite early in the test, just after class 1. This class was associated with fiber-matrix debonding (intra- and inter-yarn) and friction in the material. The dissociation of the damage mechanisms between classes 1 and 2 remained delicate because these two classes were separated in particular by their frequency content. The influence of the propagation medium and damage on the signal frequency yielded to a complex dissociation of the mechanisms. Modeling AE sources could help to understand the origin of these different sources [19,39]. It should also be noted that fiber breaks are not dissociated from other damage mechanisms. These two classes are both activated in the D3. Fiber breaks seem to be contained in classes 1 and 2 because they show a strong increase in the number of sources activated during the D3 domain, especially for class 1. The increase in class 1 and 2 sources in this stage seems to be related to the progressive breakdown of warp fibers. In addition, the increase in these low energy classes is also related to the percolation of damage (matrix cracks, intra-yarn and inter-yarn fiber-matrix debonding), leading to the macroscopic ruin of the composite.

## 4. Conclusion

An approach is proposed to merge the data from two different types of AE sensors in the classification process. Data from PicoHF and Nano30 sensors offer complementary information that helps to discriminate the different classes of AE signals. Nano30 sensor is more relevant in order to detect the early damages. Nevertheless, the use of the data collected by the PicoHF sensor increases the description of the source mainly in the frequency domain. Combining features extracted from different sensors and integrating them into single features enhances the diagnostic phase and reduces the uncertainty. This approach increases the robustness of discrimination with the aid of more complete information from multiple AE sensors. Moreover, data fusion from AE sensors with different frequency ranges allows information to be less device-dependent in order to classify signals. In addition to the statistical advantage of combining same-source data (i.e., improved estimate of a physical phenomenon via redundant observations), the use of multiple types of AE sensors may improve the accuracy of the characterization of damage mechanisms. One of the main perspectives in this field lies in the construction of more accurate data fusion in order to extract more pertinent content of the signals. This might be performed by overlapping the signals in time-frequency domain. Another approach could take into account the sensors’ characteristics in the data fusion process.

## Figures and Tables

**Table 1:** Descriptors of AE signals extracted from the waveforms.

Descriptor	Unit
Time Features	
Amplitude	dB
Duration	µs
Energy, E	attoJ
Zero-crossing rate	%
Rise time	µs
Temporal centroid	µs
Entropy	-
**Frequency Features**	
Partial Power 1 ([0; 200] kHz), PP_1 (0–200kHz)_	%
Partial Power 2 ([200; 400] kHz), PP_2 (200–400kHz)_	%
Partial Power 3 ([400; 800] kHz), PP_3 (400–800kHz)_	%
Partial Power 4 ([800; 1200] kHz), PP_4 (800–1200kHz)_	%
Centroid frequency	kHz
Peak frequency	kHz
Spectral spread	kHz
Spectral skewness	-
Spectral kurtosis	-
Roll-off frequency (90%), f_cut_	kHz
Roll-on frequency (10%), f_op_	kHz
**Combined descriptors**	
Rise time/Duration	-
Duration/Amplitude	µs·dB^−1^
Decay time (Duration –Rise time)	µs
Rise angle (Amplitude/Rise time)	dB/µs
Rise time/Decay Time	-
Energy/Amplitude	attoJ·dB^−1^
Amplitude/Centroid frequency	dB·kHz^−1^

## References

[1] Newman B., Schäfer W. (2006). Processing and Properties of Oxide/Oxide Composites for Industrial Applications.

[2] Wilson D., Visser L. (2001). High performance oxide fibers for metal and ceramic composites. Compos. Part A Appl. Sci. Manuf..

[3] Haslam J., Berroth K., Lange F. (2000). Processing and properties of an all-oxide composite with a porous matrix. J. Eur. Ceram. Soc..

[4] Levi C.G., Yang J.Y., Dalgleish B.J., Zok F.W., Evans A.G. (2005). Processing and Performance of an All-Oxide Ceramic Composite. J. Am. Ceram. Soc..

[5] Kaya C., Kaya F., Butler E.G., Boccaccini A.R., Chawla K.K. (2009). Development and characterisation of high-density oxide fiber-reinforced oxide ceramic matrix composites with improved mechanical properties. J. Eur. Ceram. Soc..

[6] Volkmann E., Tushtev K., Koch D., Wilhelmi C., Göring J., Rezwan K. (2015). Assessment of three oxide/oxide ceramic matrix composites: Mechanical performance and effects of heat treatments. Compos. Part A Appl. Sci. Manuf..

[7] Ben Ramdane C., Julian-Jankowiak A., Valle R., Renollet Y., Parlier M., Martin E., Diss P. (2017). Microstructure and mechanical behaviour of a NextelTM610/alumina weak matrix composite subjected to tensile and compressive loadings. J. Eur. Ceram. Soc..

[8] Mattoni M.A., Yang J.Y., Levi C.G., Zok F.W. (2001). Effects of Matrix Porosity on the Mechanical Properties of a Porous-Matrix, All-Oxide Ceramic Composite. J. Am. Ceram. Soc..

[9] Zok F.W., Levi C.G. (2001). Mechanical properties of porous-matrix ceramic composites. Adv. Eng. Mater..

[10] Kostopoulos V., Loutas T., Kontsos A., Sotiriadis G., Pappas Y. (2003). On the identification of the failure mechanisms in oxide/oxide composites using acoustic emission. NDT E Int..

[11] Weaver J.H., Rannou J., Mattoni M.A., Zok F.W. (2006). Interface Properties in a Porous-Matrix Oxide Composite. J. Am. Ceram. Soc..

[12] Godin N., Reynaud P., Fantozzi G. (2018). Acoustic Emission and Durability of Composite Materials.

[13] Sause M.G.R., Schmitt S., Kalafat S. (2018). Failure load prediction for fiber-reinforced composites based on acoustic emission. Compos. Sci. Technol..

[14] Loutas T., Eleftheroglou N., Zarouchas D. (2017). A data-driven probabilistic framework towards the in-situ prognostics of fatigue life of composites based on acoustic emission data. Compos. Struct..

[15] Godin N., Reynaud P., Fantozzi G. (2019). Contribution of AE analysis in order to evaluate time to failure of ceramic matrix composites. Eng. Fract. Mech..

[16] Anastassopoulos A., Philippidis T.P. (1995). Clustering methodology for the evaluation of acoustic emission from composites. J. Acoust. Emiss..

[17] Godin N., Reynaud P., Fantozzi G. (2018). Challenges and Limitations in the Identification of Acoustic Emission Signature of Damage Mechanisms in Composites Materials. Appl. Sci..

[18] Le Gall T., Monnier T., Fusco C., Godin N., Hebaz S.E. (2018). Towards Quantitative Acoustic Emission by Finite Element Modelling: Contribution of Modal Analysis and Identification of Pertinent Descriptors. Appl. Sci..

[19] Hamam Z., Godin N., Fusco C., Monnier T. (2019). Modelling of Acoustic Emission Signals Due to Fiber Break in a Model Composite Carbon/Epoxy: Experimental Validation and Parametric Study. Appl. Sci..

[20] Luo R.C., Yih C.C., Su K.L. (2002). Multisensor fusion and integration: Approaches, applications, and future research directions. IEEE Sens. J..

[21] Zhang R., Nie F., Li X., Wei X. (2019). Feature selection with multi-view data: A survey. Inf. Fusion.

[22] Llinas J., Hall D. (2002). An Introduction to Multi-Sensor Data Fusion.

[23] Gros X.E. (2001). Multisensor Data Fusion and Integration in NDT. Applications of NDT Data Fusion.

[24] Eleftheroglou N., Zarouchasa D., Loutas T., Alderliesten R., Benedictus R. (2018). Structural health monitoring data fusion for in-situ life prognosis of composite structures. Reliab. Eng. Syst. Saf..

[25] Yang J., Yang J.-Y., Zhang D., Lu J.-F. (2003). Feature fusion: Parallel strategy vs. serial strategy. Pattern Recognit..

[26] Morscher G.N., Gyekenyesi A.L. (2002). The velocity and attenuation of acoustic emission waves in SiC/SiC composites loaded in tension. Compos. Sci. Technol..

[27] Hatano H. (1976). Acoustic-emission transducer and its absolute calibration. J. Acoust. Soc. Am..

[28] Dia S., Monnier T., Godin N., Zhang F. (2012). Primary Calibration of Acoustic Emission Sensors by the Method of Reciprocity, Theoretical and Experimental Considerations. J. Acoust. Emiss..

[29] Goujon L., Baboux J.C. (2003). Behaviour of acoustic emission sensors using broadband calibration techniques. Meas. Sci. Technol..

[30] Morizet N., Godin N., Tang J., Maillet E., Frégonèse M., Normand B. (2016). Classification of acoustic emission signals using wavelets and Random Forests: Application to localized corrosion. Mech. Syst. Signal Process..

[31] Fowler T.J. (1979). Acoustic emission of fiber reinforced plastics. J. Tech. Counc. ASCE.

[32] Pohl C., Van Genderen J.L. (1998). Review article Multisensor image fusion in remote sensing: Concepts, methods and applications. Int. J. Remote. Sens..

[33] D’Costa A., Sayeed A.M. (2003). Collaborative Signal Processing for Distributed Classification in Sensor Networks. Information Processing in Sensor Networks.

[34] Moevus M., Godin N., R’Mili M., Rouby D., Reynaud P., Fantozzi G., Farizy G. (2008). Analysis of damage mechanisms and associated acoustic emission in two SiCf/[Si–B–C] composites exhibiting different tensile behaviours. Part II: Unsupervised acoustic emission data clustering. Compos. Sci. Technol..

[35] Davies D.L., Bouldin D.W. (1979). A Cluster Separation Measure. IEEE Trans. Pattern Anal. Mach. Intell..

[36] Rousseeuw P.J. (1987). Silhouettes: A graphical aid to the interpretation and validation of cluster analysis. J. Comput. Appl. Math..

[37] Sibil A., Godin N., R’Mili M., Maillet E., Fantozzi G. (2012). Optimization of Acoustic Emission Data Clustering by a Genetic Algorithm Method. J. Nondestruct. Eval..

[38] Caty O., Mazars V., Bertrand R., Denneulin S., Couegnat G., Vignoles G. Application of X-Ray computed micro-tomography to the study of damage, self healing and oxidation of thermostructural composite. Proceedings of the 17th European Conference on Composite Materials.

[39] Sause M.G.R., Horn S. (2010). Simulation of Acoustic Emission in Planar Carbon Fiber Reinforced Plastic Specimens. J. Nondestruct. Eval..

